# Enhancing Robotic Antenna Measurements with Composite-Plane Range Extension and Localized Sparse Sampling

**DOI:** 10.3390/s25237200

**Published:** 2025-11-25

**Authors:** Celia Fontá Romero, Ana Arboleya, Fernando Rodríguez Varela, Manuel Sierra Castañer

**Affiliations:** 1 Information Processing and Telecommunications Center, Radiation Group, Department of Signals, Systems and Radiocommunications, Universidad Politécnica de Madrid, E-28040 Madrid, Spain; manuel.sierra@upm.es; 2 Group of Microwave Engineering and Radiocommunication Systems, Department of Signal Theory and Communications, Universidad Rey Juan Carlos, E-28942 Madrid, Spain; ana.arboleya@urjc.es (A.A.); fernando.varela@urjc.es (F.R.V.)

**Keywords:** antenna testing, composite-plane, fast measurements, in situ measurements, near-field, portable systems, range extension, robotic measurements, singular value decomposition, sparse sampling

## Abstract

**Highlights:**

**What are the main findings?**

- Portable robotic antenna measurement framework integrating range extension techniques with localized, SVD-based sparse sampling for angular sectors of interest.
- Larger effective scan area than the robot’s reach and fewer samples in target sectors, while accuracy is maintained.

**What is the implication of the main finding?**

- Practical approach to employ medium-size robots for in situ characterization of electrically large or tilted-beam antennas without complex systems or intricate calibrations.
- Demonstrates that the proposed approach can decouple system reach from time-to-measure, informing the design of future cost-effective, portable and accurate robotic antenna metrology setups.

**Abstract:**

Robotic arm-based antenna measurement systems offer the flexibility needed for advanced antenna measurement and diagnostics techniques but are typically limited by reach and sampling time. This work integrates two complementary contributions to overcome these constraints. First, a composite-plane range extension is introduced for a medium-size robot mounted on a mobile platform and monitored by an optical tracking system (OTS). Independent planar scans are acquired after manual repositioning of the robot and then accurately aligned and blended into a single, larger measurement plane, with positioning errors mitigated through a calibration process. Second, a localized sparse sampling strategy is proposed to accelerate planar near-field (PNF) measurements when only selected angular regions of the radiation pattern are required. The approach relies on reduced-order modeling and singular value decomposition (SVD) analysis to design non-redundant grids that preserve the degrees of freedom relevant to the truncated angular sector, thereby reducing both the number of samples and the scan area. Numerical examples for a general case and experimental validation in X-band demonstrate that the combined methodology extends the effective measurement aperture while significantly shortening acquisition time for narrow or tilted beams, enabling accurate and portable in situ characterization of complex modern antennas by means of cost-effective acquisition systems.

## 1. Introduction

Antenna technologies for emerging wireless systems continue to grow in electrical size and architectural complexity, from multi-beam panels [1,2] to large reconfigurable apertures [3,4], including broadband antennas [5,6], large or platform integrated antennas [7,8], etc. Their characterization demands high-frequency, (ever more) portable systems for in situ measurements and advanced sampling schemes that exceed the practical limits of conventional anechoic chambers and fixed scanners [9].

Portable solutions based on unmanned aerial vehicles (UAVs) or handheld probes have been explored; UAVs enable large outdoor scans but face positioning-accuracy limits at high frequencies [10,11], often requiring laser-tracker augmentation [12,13], while handheld systems remove mechanical constraints at the cost of continuous human intervention and time-intensive operation [14,15].

In parallel, robotic-arm-based systems have consequently gained interest as a versatile alternative, supporting conventional geometries and non-standard trajectories with high accuracy and reduced mechanical constraints [16,17]. Nevertheless, three persistent obstacles remain: (i) restricted range of motion, which limits the accessible scan area; (ii) many existing robotic-arm installations are fixed in anechoic environments and, thus, are not readily portable for in situ campaigns; and (iii) the acquisition burden of dense sampling over wide angular regions when only a subset of the pattern is required, an issue inherent to electrically large antenna measurements with conventional sampling techniques.

A practical route to mitigate the restricted range of motion is to extend the workspace mechanically, either by coupling the arm with auxiliary positioners (e.g., linear sliders or additional stages) [18,19,20] or by coordinating multiple robotic arms [21,22]. These approaches are effective, but increase complexity and cost, and may constrain the antenna under test (AUT) size when mounting is required on a moving axis. Recent developments have explored portable robot-based configurations [23,24] that preserve flexibility without heavy mechanics, leveraging tracking systems for increased accuracy or range extension [25].

Regarding sampling schemes, non-redundant strategies and reduced-order formulations alleviate the acquisition time in near-field systems. Analytical non-redundant grids bound the field degrees of freedom on the scan surface and, together with optimal sampling interpolation (OSI), enable accurate reconstructions from minimum datasets [26,27,28,29,30]. Complementarily, singular value decomposition (SVD) formulations cast the NF–FF transformation as an inverse problem, allowing reduced AUT models, conditioning control, and the optimization of measurement layouts compatible with raster or multi-probe acquisitions [31,32,33,34,35]. In practice, when the application targets a truncated angular region (e.g., a narrow or strongly tilted main lobe), localized sparse designs confine the unknowns to the degrees of freedom that effectively contribute within that sector, which concentrates measurements on information-rich areas and reduces both sample count and scan-plane size while preserving accuracy [36,37,38,39,40].

This work addresses the limitations mentioned above by combining a composite-plane range extension [25] with a localized sparse sampling methodology for fast planar near-field (PNF) measurements [40]. The system is implemented by means of a medium-size collaborative robot mounted on a portable base and instrumented with an optical tracking system (OTS). The OTS provides a global coordinate reference to align multiple partial scans acquired after manually repositioning the platform. A simple calibration procedure compensates robot and probe displacements, fusing the segments into a smooth, enlarged measurement surface and removing restricted range of motion limitations. On the sampling side, the fact that many applications require accurate knowledge of the radiated field only within a specified angular sector (i.e., around a main lobe with significant tilt) is exploited. A localized inverse problem whose unknowns are limited to the degrees of freedom that effectively contribute to that truncated region is formulated. Singular-value analysis then guides a reduced-order representation and the design of non-redundant grids compatible with raster scanning. The result is a sparse acquisition strategy that concentrates measurements where information is most valuable, cutting both sample count and scan plane size while maintaining accuracy for most applications. The proposed approach has been validated through numerical examples and experimentally for a general case and X-band respectively.

The rest of the manuscript is structured as follows: Section 2 describes the methodology for composite-plane acquisition, near-field to far-field transformation and grid-reduction techniques; Section 3 presents several numerical validation examples in a general case as a function of the wavelength; Section 4 gathers the main results from the experimental validation in the X-band including an extensive positioning error analysis; finally, Section 5 concludes the manuscript with a discussion of the obtained results.

## 2. Methodology

The primary objective of this work is to present the implementation and integration of two proposed techniques. The composite-plane technique [25] enables the portable system to generate a precisely defined planar surface by combining multiple individual planes that the system can physically sweep (scan size within robot’s reach). This approach is integrated with a grid-reduction technique [40]. However, the integration of both techniques introduces challenges for the composite-plane due to the critical role of sampling in the sparse designed grid. Consequently, system precision becomes significantly more crucial than when employing a conventional acquisition surface (i.e., single-plane acquisition). The aim of this paper is to conduct a general-case study, in terms of the wavelength, to evaluate the applicability of reduced grids in composite-plane measurement techniques and to quantify the error introduced in the recovered far-field pattern when non-redundant sampling is employed. The reduced grid is applied to the composite-plane technique without any additional considerations, ensuring a fair evaluation and allowing the study to reveal whether extra measurements or considerations are required.

In this section, a brief discussion about the two implemented techniques will be presented.

### 2.1. Composite-Plane Technique

As previously established, the main objective is range extension of the measurement system through plane composition. In the case addressed in this work, the employed system is a portable setup consisting of a robotic arm mounted on a hydraulic platform, which allows for easy displacement. Since the displacement of the robotic arm is not motorized or supported by a precision mechanism, it must be performed manually. Consequently, significant positioning errors may arise due to human interaction. To mitigate this effect, an OTS based on infrared (IR) cameras is employed.

The camera subsystem continuously tracks the position of the robot’s tool flange (where the antenna probe is installed, with several reflective markers attached) throughout the measurement process. This tracking enables the compensation of alignment errors between the AUT and the probe, as well as the calibration of the system to determine the exact position and orientation of the individual planes. Once the robot is manually displaced to (approximately) the target position, i.e., the center of one of the individual planes, the system computes the necessary correction and automatically applies it to the robot to ensure accurate alignment. See [25] for more detailed information.

The accuracy of the OTS depends on the number and placement of the IR cameras which define the working volume. Marker placement is optimized to maximize visibility and minimize occlusions (the manufacturer recommends that each marker be visible to at least three cameras). Tracking accuracy can be increased by increasing the number of IR cameras and/or reducing the working volume or by means of active markers. Different camera layouts and marker placements were tested to ensure robust tracking at high frequencies. The current system configuration includes four IR cameras that achieve sub-millimeter accuracy within a working volume of approximately 4–5 m in diameter and eight 8 mm diameter spherical markers mounted on a 3D-printed fixture attached to the probe.

It is worth noting that the IR cameras are sensitive to strong ambient IR sources (e.g., direct sunlight or unfiltered IR lighting), therefore, when used outdoors, measurements should be performed away from direct solar illumination or outside peak daylight hours. Also, highly reflective surfaces or nearby metallic structures can produce spurious IR reflections; the measurement setup must be arranged to minimize such surfaces in the tracking volume whenever possible.

**Figure 1:**
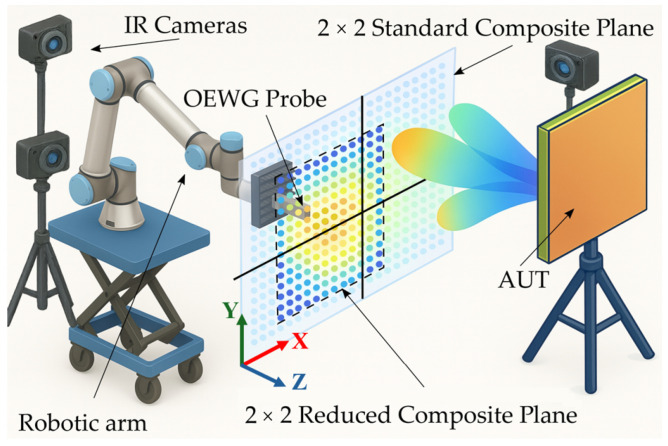
Scheme of the robotic-arm system setup for a 2×2 composite-plane.

A general overview of the system is shown in Figure 1. The setup consists of a UR10e robotic arm from Universal Robots (Odense, Denmark) mounted on a hydraulic scissor platform. Four IR cameras from OptiTrack (Corvallis, OR, USA) are installed within the perimeter of the working volume in configurations optimized for tracking the robot’s location according to the measurement requirements. The probe antenna, a rectangular open-ended waveguide (OEWG), is mounted on the robot’s tool flange using a custom 3D-printed fixture, while the AUT is mounted on a tripod. In this figure, a 2×2 composite-plane is illustrated. The black lines denote the boundaries between the individual planes that the robot can physically sweep. The figure shows a reduced grid superimposed on a conventional regular planar grid. In both cases, the composite plane is required.

Measurements are performed in the near-field to achieve the widest possible angular validity. Once the plane size and the distance from the AUT are determined, the grid is defined. The objective of these near-field measurements is to subsequently reconstruct the far-field pattern. Since the acquisition is performed on a planar surface, the technique employed to obtain the farfield relies on the plane wave spectrum (PWS) formulation given in (1), where $\vec{P}$ and $\vec{E}$ denote the PWS and the aperture field, respectively, *k* is the wavenumber, *x* and *y* are the Cartesian coordinates of the aperture (located at z=0), and *u* and *v* are the angular coordinates defined such that $u^{2}+v^{2}\leq 1$:

(1) $$\vec{P}\left(u,v\right)=\int \int_{S}\vec{E}\left(x,y\right)e^{jk(ux+vy)}dxdy.$$

However, the employed sampling grid is non-uniform, due to the use of actual coordinates provided by the OTS instead of nominal grid coordinates (and, in the case of this work, due to the use of a sparse irregular grid), therefore, (1) cannot be applied directly. To address this issue, a matrix inversion technique is proposed [40]. In this formulation, the aperture field $\vec{E}\left(x,y\right)$ is collected in a vector $e_{a}$, which can be related to the vector $e_{m}$ containing the samples of the measured field through a plane-to plane transformation matrix F, according to (2):

(2) $$e_{m}=Fe_{a}=TMe_{a}$$

In (2), $e_{m}$ is defined as the concatenation of all the measured individual planes, where the OTS coordinates (defined on the sparse irregular grid) are incorporated into matrix F. This matrix contains both transformations: M is the matrix operator performing the numerical integration in (1) and T performs the transformation between the PWS and the planar field. For clarity of exposition, probe correction is not included in the formulation presented here. This step was also omitted in the processing of the measured fields, as it does not affect the relative validation of the proposed composite-plane and reduced-grid technique. Nevertheless, the presented techniques are compatible with standard probe correction methods as is described in [40].

Once the aperture field $e_{a}$ is obtained by solving the inverse problem in (2), the corresponding far-field can be readily derived from the PWS computed from this reconstructed field as expressed in (3):

(3) $$p=Me_{a},$$

where p is a vector containing the samples of $\vec{P}\left(u,v\right)$ in the angular region of the far-field pattern of interest.

### 2.2. Reduced Angular Pattern Domain Technique

The concept of this technique, developed in [40], is to reduce the number of required sampling points by focusing only on the spectral information that is strictly necessary. In many practical scenarios, only a specific region of the far-field is of interest. In such cases, it is not necessary to perform a complete acquisition if full spectral information is not required.

The reduction introduced by this approach relies on two main considerations. First, the transformation matrices are defined only within the reduced angular region of interest. This results in fewer equations needed to determine the aperture field which completely characterizes the antenna. Consequently, the transformation matrices M and T are also reduced, yielding a simplified version of the complete transformation matrix for the inverse problem.

Second, a truncation is applied to the singular values of the matrix decomposition. Specifically, the relationship between the aperture field $e_{a}$ and the truncated PWS $p_{r}$.

An SVD of M factorizes it into three matrices, $M=USV^{H}$, where U and V are unitary matrices containing the left and right singular vectors, respectively, S is a diagonal matrix containing the singular values and ${\left(\cdot \right)}^{H}$ denotes the Hermitian operator. The singular values in S quantify the relative importance of each vector in reconstructing the spectrum. The number of equations involved in the inverse-problem can be reduced by truncating the vectors in V that correspond to very small singular values (i.e., those with negligible contribution to the spectrum) as described in [40]. In this work, the truncation threshold has been chosen to discard contributions below the system noise level, which is −50 dB. Such truncation significantly reduces the number of equations required for solving the inverse problem without affecting the aperture-field recovery, as no relevant information is discarded. Greater reduction can be obtained through more aggressive truncation, although this may lead to the loss of potentially meaningful contributions. For this reason, the optimal choice depends on the measurement system and the balance between desired reduction and reconstruction accuracy. As a result, the reduced matrices $S_{r}$ and $V_{r}^{H}$ are obtained. The truncation also decreases the number of coefficients needed to compute $e_{a}$ while preserving the most relevant spectral content:

(4) $$p_{r}\approx US_{r}V_{r}^{H}e_{a}.$$

This reduced equation system, in which the number of unknowns is significantly decreased, can be further optimized by determining the reduced grid. Some calculations can be performed prior to measurement to determine the optimal grid depending on some initial parameters such as the number of desired points (that will be analyzed in the following section) and which kind of grid is desired. In this case, Legendre polynomials are used to define the grid, so the number of variables are reduced to the coefficients of the polynomials. This is further explored in [40]. This can be formulated as an optimization problem, where the goal is to compute a reduced grid that ensures an accurate reconstruction of the values.

## 3. Numerical Example

Before experimental validation of the proposed methodology, several numerical simulations were performed for a general case, in which all parameters are expressed in terms of wavelength λ, rather than a fixed operating frequency. This is based on the experiments performed in [41].

A parabolic reflector was simulated using GRASP (10.6.1 version, 2017) from TICRA (Copenhagen, Denmark). The reflector exhibits a λ offset in the Y-axis and a Gaussian illumination taper of −12 dB. The aperture size is 10λ with an F/D ratio of 0.5, where *F* denotes the focal length and *D* the diameter of the reflector. This configuration was selected as a representative case that allows the methodology to be evaluated under controlled yet realistic conditions.

The objective of this numerical study is to analyze the impact of different mechanical acquisition errors on the reconstruction of a reduced angular region of the spectrum, when applying the technique described in the previous sections. In addition, several candidate optimal grids are investigated in order to better understand the trade-off between grid-reduction factor and sensitivity to acquisition errors. These results provide valuable insight prior to the experimental validation, establishing the expected limitations and performance trends of the method.

**Figure 2:**
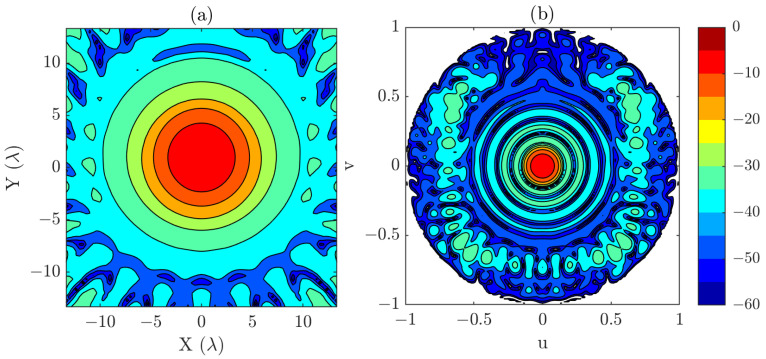
(**a**) Simulated near-field (dB). (**b**) PWS obtained from simulated near-field (dB). Those results are considered the reference results for the numerical examples.

In Figure 2, both the near-field distribution and the corresponding PWS are presented. Those fields have been computed from a uniform grid of 54×54 points and λ/2 sampling step and are used as the reference results. Although the simulated field is relatively simple, the measurement distance of 15λ was deliberately chosen so that the resulting beam covers a large area. This condition recreates a scenario in which the use of a composite-plane acquisition could become necessary to fully capture the relevant information.

The region of interest in the PWS that will be reconstructed in the tests with the reduced grids is defined as −0.25<u<0.25 and −0.25<v<0.25, which corresponds to the angular span of the main beam (see Figure 2b). Focusing on this reduced area allows us to evaluate the effectiveness of the proposed methodology in recovering the most significant spectral content.

Simulation results will be processed to evaluate the errors associated with performing the acquisition on a 2×2 composite plane. The total acquisition surface is 26.5λ×26.5λ, measured at a distance of 15λ. Some OTS data from real measurements are used to introduce the positioning error distribution, which is weighted to simulate errors ranging from λ/8 to λ/2. Results obtained using both the OTS coordinates and the nominal coordinates are shown for three different reduced acquisition grids, each with a different reduction factor. The objective of this section is to analyze the frequency limitations of the technique and their impact on the reduction factor.

As previously mentioned, the objective is to introduce different acquisition errors in order to analyze their impact on the recovery of the spectrum. For this purpose, real OTS coordinate data obtained from robotic-arm acquisitions have been numerically introduced into the simulation results, scaled to preserve the actual error distribution of the system while varying its magnitude. These errors have been separated into three components: X-axis, Y-axis, and Z-axis deviations, where X and Y correspond to the acquisition coordinates (with the sweep performed along the Y-axis), and Z represents the acquisition plane distance (see Figure 1 for axes definition).

**Figure 3:**
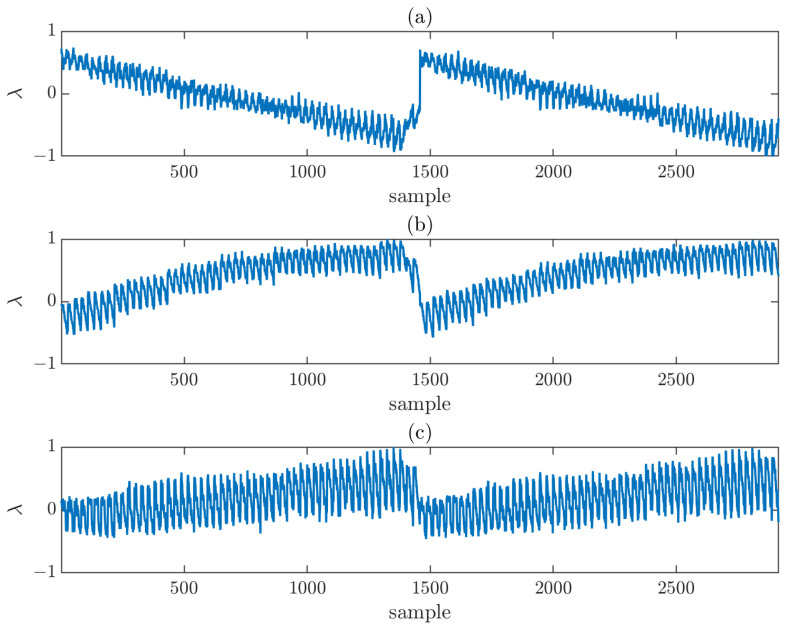
Normalized error distribution (in wavelengths, λ) for each sample of the acquisition surface, along each axis: (**a**) X-axis, (**b**) Y-axis, (**c**) Z-axis.

To ensure a controlled analysis, the errors were normalized and subsequently weighted to adjust the maximum error introduced in each acquisition. This procedure allows the influence of each component to be independently assessed while keeping the overall error contribution within predefined limits. The normalized error distributions for each axis are shown in Figure 3 considering a 2×2 composite-plane of the same size as the reference simulation (i.e., 54×54 points with λ/2 sampling) that will be referred to as N = 54. It is interesting to observe the effect around sample 1500, corresponding to the transition between the left and right individual planes, where a pronounced error is noticeable. The transition between the top and bottom individual planes appears smoother in the representation because the sweep axis is the Y-axis.

Following the methodology described in the previous section, three different reduced grids have been computed as described in [40]. These grids contain 6×6 (98.8% reduction factor), 9×9 (97.2% reduction factor), and 12×12 (95% reduction factor) sampling points, and will be referred to as N=6, N=9, and N=12, respectively from now on. This selection enables a direct comparison of the effect of grid density on the reconstruction accuracy of the spectrum. All cases are expected to enable proper recovery, as indicated by the condition number and eigenvalues distribution of the F matrix. The case with N = 6 represents a significant reduction that still preserves the recovery capability, while N = 12 yields recovery properties comparable to those of a full acquisition.

**Figure 4:**
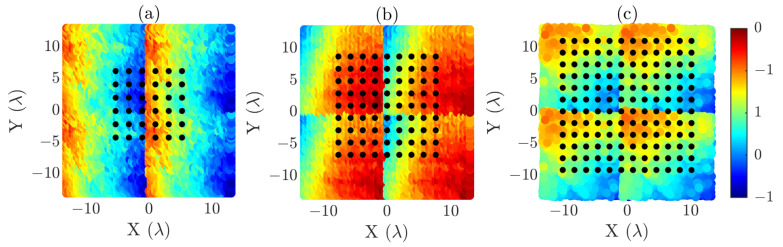
Optimized acquisition coordinates overlaid on the normalized error distribution of the acquisition plane. The error distribution is shown separately along each axis in the subfigures, but the underlying distribution is the same for all three acquisition grids. (**a**) N = 6 reduced grid and X-axis error (in λ), (**b**) N = 9 reduced grid and Y-axis error (in λ), (**c**) N = 12 reduced grid and Z-axis error (in λ).

To illustrate the spatial distribution of these grids, Figure 4 presents three graphs in which the acquisition coordinates captured by the OTS, including the normalized acquisition errors, are represented together with the corresponding reduced-grid overlays. Figure 4a–c represent the X-, Y-, and Z-axis errors, respectively. In each case, one of the three reduced grids is superimposed. It is important to emphasize that the error distribution is the same for all reduced-grid cases; they are shown separately only for representational purposes. In addition, the reduced grids are also subject to acquisition-plane errors introduced during the measurement process.

Let ϵ be defined as the maximum error introduced in the error distribution. Accordingly, the acquisition error along each axis is normalized with respect to this factor. For the numerical validation presented in this section, three different maximum error values have been considered: ϵ=λ/8, ϵ=λ/4, and ϵ=λ/2. These values were selected to represent progressively more demanding error conditions, ranging from relatively small perturbations to severe misalignments.

**Figure 5:**
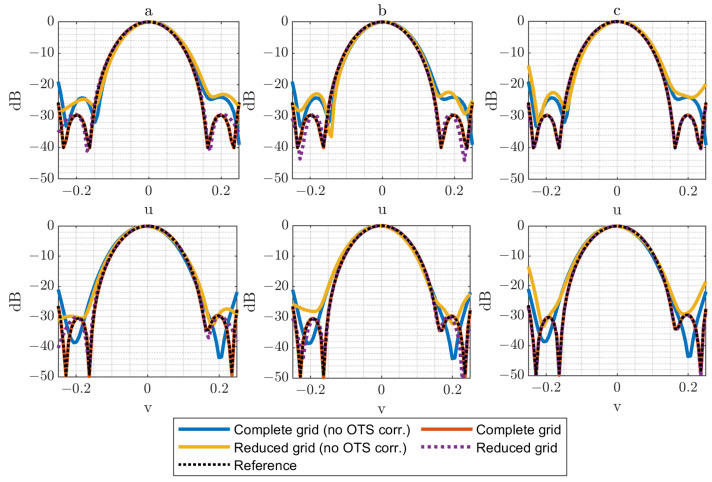
Horizontal and vertical cuts of the PWS (dB) for ϵ=λ/8 for complete (N = 54) and reduced grids: (**a**) N = 6 reduced grid, (**b**) N = 9 reduced grid, (**c**) N = 12 reduced grid.

**Figure 6:**
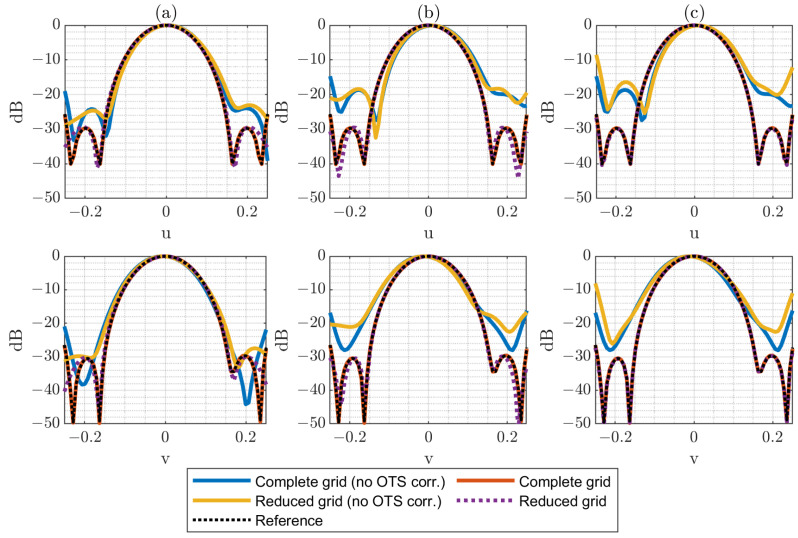
Horizontal and vertical cuts of the PWS (dB) for ϵ=λ/4 for complete (N = 54) and reduced grids: (**a**) N = 6 reduced grid, (**b**) N = 9 reduced grid, (**c**) N = 12 reduced grid.

**Figure 7:**
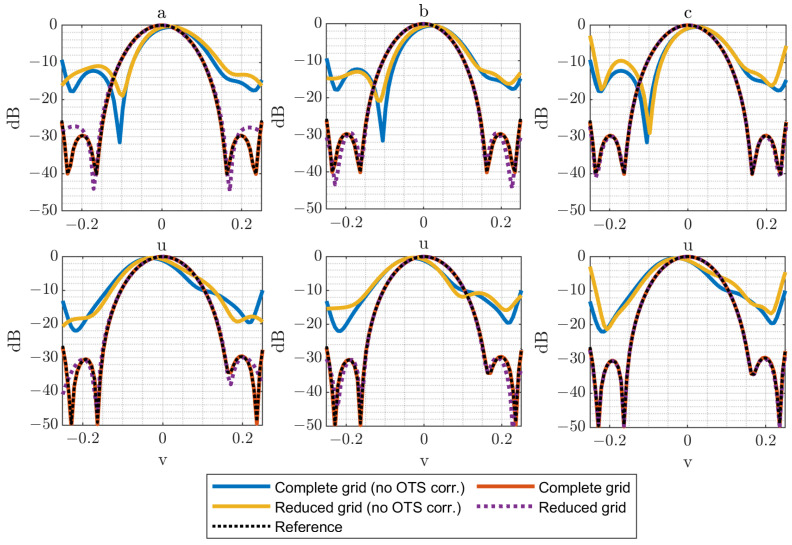
Horizontal and vertical cuts of the PWS (dB) for ϵ=λ/2 for complete (N = 54) and reduced grids: (**a**) N = 6 reduced grid, (**b**) N = 9 reduced grid, (**c**) N = 12 reduced grid.

In Figure 5, Figure 6 and Figure 7, several plots are displayed to illustrate the effect of these error levels. Specifically, both horizontal and vertical cuts of the spectrum are presented, allowing a direct comparison between the complete acquisition grid and the reduced acquisition grids. The results are presented for cases where either the OTS-provided coordinates are considered or the nominal coordinates are used (no OTS corr.). This comparison highlights the importance of accounting for the OTS coordinates (i.e., real acquisition points) instead of the nominal grid (i.e., ideal acquisition points) when applying the reduced-grid technique, particularly with larger acquisition errors. The reference used for comparisons corresponds to the PWS obtained from a full regular measurement without positioning error distribution.

To quantify the error between the obtained spectrums, the merit figure η is defined as in (5), where $P_{i}$ denotes the PWS whose error is to be quantified, $P_{r}$ is the reference, and *n* indexes the samples (out of a total of $N_{s}$ samples). The quantity is expressed as a percentage, based on the error definition in [41]. The errors obtained from each case can be seen in Table 1, Table 2 and Table 3.

(5) $$\eta =100\cdot \frac{\sum_{n=1}^{N_{s}}{\left|P_{i}\left[n\right]-P_{r}\left[n\right]\right|}^{2}}{\sum_{n=1}^{N_{s}}{\left|P_{i}\left[n\right]\right|}^{2}}$$

The effectiveness of the OTS-based correction is remarkable across the different scenarios, both for the complete and the reduced grids. This correction proves to be critical, since the retrieved PWS can vary drastically when the true acquisition positions are not taken into account. It is also noteworthy that the reduced grids reproduce the results of the complete acquisition with high fidelity and that this agreement improves as the number of samples in the reduced grid increases. Given that the acquisition field corresponds to a relatively wide beam, the technique clearly benefits from the use of denser reduced grids.

Nevertheless, the impact of acquisition errors becomes more evident when larger deviations are introduced (λ/2 in Figure 7). This effect is especially noticeable in the N=6 case, where the limited number of samples makes the reconstruction more sensitive to the assumption of an error-free acquisition surface in the calculations. Also, for the N=12 cases, it is noticeable that the error increases considerably when OTS data are not used. This is explained by the larger grid, which is more affected by regions of the composite plane with a higher error distribution.

It should also be emphasized that, in practical measurement scenarios, the accuracy of the spectrum recovery will ultimately be limited by the intrinsic errors of the OTS, which typically range between 100 and 400 μm (data provided by OptiTrack proprietary software Motive 3.1.0 v. in each calibration) depending on the operating conditions. However, these values remain sufficiently small to allow the proposed methodology to be effectively applied over a wide range of frequencies, thus preserving the applicability of the approach in realistic experimental setups.

## 4. Experimental Validation at 10 GHz

As discussed in the previous sections, the main limitation of the proposed approach stems from robot-intrinsic pose errors (e.g., gearbox backlash, joint compliance, thermal drift, etc.) together with composite-plane acquisition. Although OTS-based referencing and post-processing reduce these effects, the residual errors are non-negligible, especially for grid-reduced acquisitions, where accuracy depends on the exact spatial placement of the computed sampling points. Small offsets manifest as phase inconsistencies, degrading the reconstructed field. In composite-plane operation, the impact accumulates because each individual plane contributes its own positioning error; robust composition therefore requires highly consistent positioning throughout the measurement campaign.

In order to evaluate the effect of those errors, this section presents a comparison between measurements obtained with a single-plane acquisition (reference measurement) and those derived from the composite-plane approach. This comparison allows to explicitly quantify the influence of positioning errors on both methodologies. To further highlight the limitations of the system, a “worst-case” scenario has been intentionally designed: The individual planes are close to the maximum area that the robot can physically sweep (i.e., 700 mm × 900 mm for the implemented setup). Under these conditions, positioning errors are maximized, particularly at the edges of the scanned region and in the transition zones between adjacent planes, which are precisely the most sensitive parts in the composite-plane reconstruction. So, in this section, the PWS obtained for the following cases is going to be analyzed:

- Complete single-plane acquisition (regularly sampled).
- Complete 2×2 composite-plane acquisition (regularly sampled).
- Reduced grid for single-plane acquisition.
- Reduced grid for 2×2 composite-plane acquisition.

To carry out this analysis, the complete acquisition grids are first defined, together with the corresponding coordinates provided by the OTS, in order to characterize the positioning errors associated with the measurement process. Then, the optimized grid is computed, and the PWS obtained from it is presented in comparison with that obtained from the complete acquisition.

**Figure 8:**
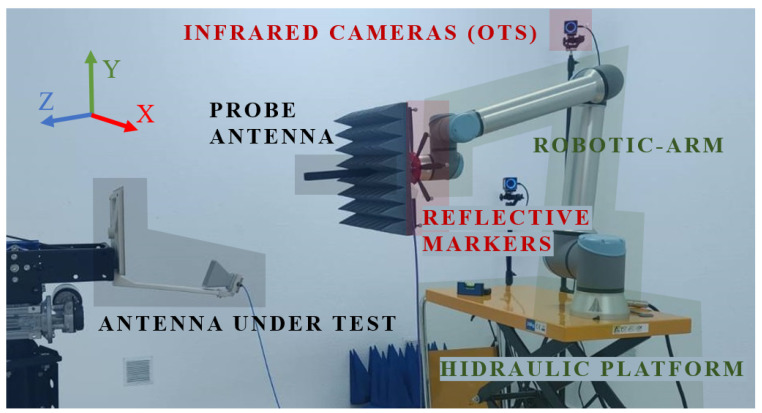
Measurement setup for single-plane and 2×2 composite-plane acquisitions. The main system components are indicated in different colors.

The AUT selected for these experiments is a reflectarray operating at 10 GHz, with physical dimensions of 200 mm × 200 mm with its main beam pointing towards (u,v)=(0,−0.4). For the single-plane acquisition case, a measurement area of 700 mm × 900 mm is considered. In contrast, when applying the composite-plane approach, the effective acquisition surface is extended to a total of 1400 mm × 1400 mm (using a 2×2 composite-plane with 700 mm × 700 mm planes). The measurement setup can be seen in Figure 8.

Typical on-site deployment time is about 1 h. The installation and calibration of the OTS constitute the most time-consuming step, followed by securing and leveling the robot platform when the floor is uneven. The cameras and tripods are lightweight and single-person manageable. In terms of mass and transportability, the robot weighs 33.5 kg, the VNA 40 kg (replaceable by handheld units when dynamic-range requirements allow), and the hydraulic platform approximately 100 kg, which is currently the primary constraint for outdoor logistics. On-site operation requires clear space to place the robot on its platform and to arrange the IR cameras so as to define a working volume that fully covers the intended measurement plane. In space-constrained sites, truncated acquisition surfaces may be necessary, which can degrade measurement quality and narrow the validity range of the computed far-field.

**Figure 9:**
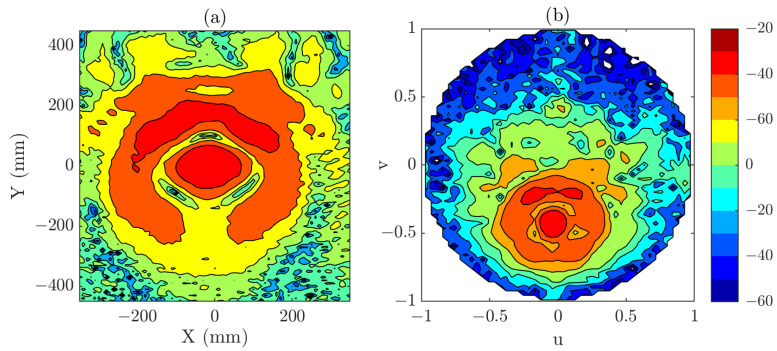
(**a**) Measured field in the single plane acquisition (dB). (**b**) PWS obtained from the single plane acquisition (dB). Those results are considered the reference results for the experimental validation example.

Figure 9 illustrates both the measured near-field distribution and the corresponding PWS obtained for the single-plane acquisition, which serves as the reference case for subsequent evaluations. In this experiment, the reduced angular region to be reconstructed using the proposed technique is defined as −0.3<u<0.3 and −0.7<v<−0.1.

**Figure 10:**
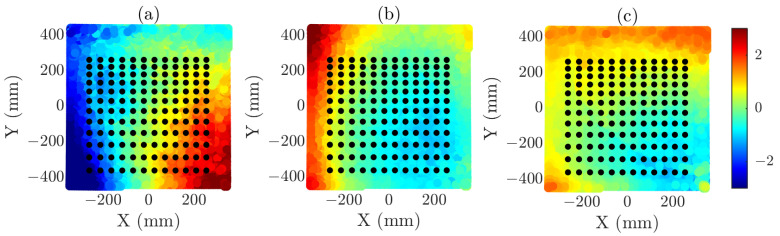
Optimized acquisition coordinates overlaid on the normalized error distribution of the acquisition plane. The error distribution is shown separately along each axis in the subfigures. (**a**) X-error in single-acquisition plane (mm). (**b**) Y-error in single-acquisition plane (mm). (**c**) Z-error in single-acquisition plane (mm). Black grid represents the reduced acquisition grid.

**Figure 11:**
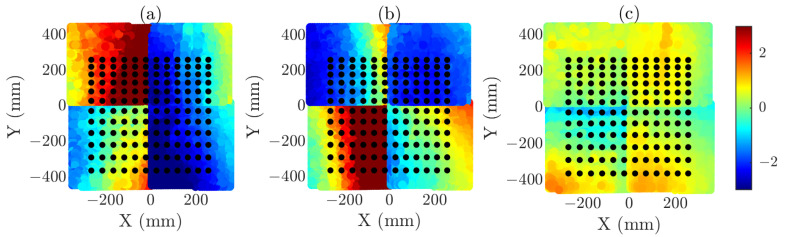
Optimized acquisition coordinates overlaid on the normalized error distribution of the acquisition plane. The error distribution is shown separately along each axis in the subfigures. (**a**) X-error in composite acquisition plane (mm). (**b**) Y-error in composite acquisition plane (mm). (**c**) Z-error in composite acquisition plane (mm). Purple grid represents the reduced acquisition grid.

Once the desired angular region for the calculations is defined, the sampling grid is generated using an iterative procedure (detailed in [40]). In this case, a 12×12 point grid has been used to determine the optimal sampling positions, compared to the samples 71×91 for the complete regularly sampled single plane (97.8% reduction) and the samples 141×141 for the composite plane (99.3% reduction). The difference in reduction factors arises from the larger size of the composite-plane. Figure 10 and Figure 11 illustrate the resulting reduced grid superimposed on the error distribution of the measurement grid captured by the cameras, in which the error for each individual axis is represented.

As can be seen, positioning errors are relatively large. This effect arises from several factors, the most critical being the physical size of the scanned plane, since larger areas are strongly correlated with increased positioning inaccuracy. In the case of the composite-plane acquisition, the junction regions between adjacent planes further contribute to the observed errors, creating a large error between adjacent samples in the central regions of the composite-plane.

**Figure 12:**
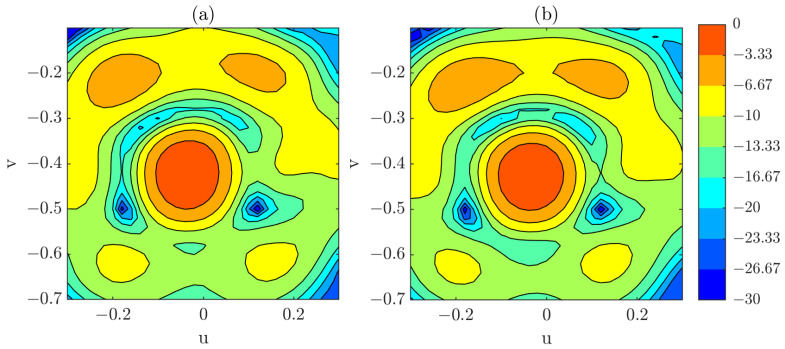
(**a**) PWS obtained from a full, regularly sampled single-plane acquisition (dB). (**b**) PWS obtained from a full, regularly sampled composite-plane acquisition (dB).

Within the defined angular interval, a comparison is carried out between the PWS obtained from the complete single-plane acquisition and that obtained from the complete composite-plane acquisition. For both cases, a coordinate-correction procedure is applied during post-processing to ensure proper alignment of the reconstructed fields. PWS results when using a complete and regularly sampled grid acquisition for the defined angular span can be observed in Figure 12. The differences between both spectrums are minor within the considered region, which suggests that the composite-plane approach is capable of reproducing the PWS for a regularly sampled and complete measurement. It is important to note that in both cases an OTS-coordinates have been used, as was done in the previous section.

**Figure 13:**
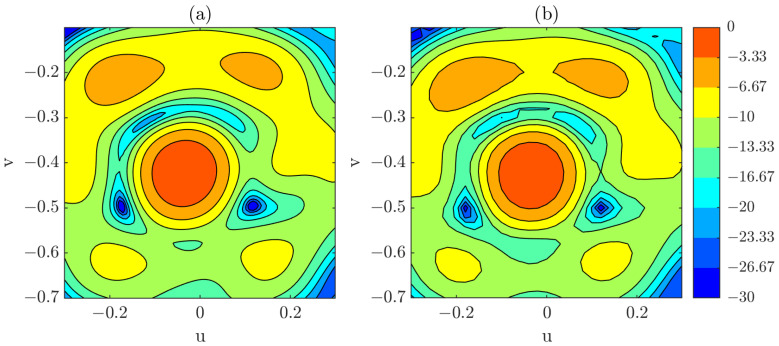
(**a**) PWS obtained from the reduced grid using the composite-plane acquisition (dB). (**b**) PWS obtained from a full, regularly sampled single-plane acquisition (dB).

After the test for the complete measurements yielded satisfactory results, the focus is now placed on the reduced acquisitions, both for single- and composite-plane cases. The results from the complete, regularly sampled single-plane acquisition are used as the reference for the subsequent analysis. A visual comparison is presented Figure 13, where the PWS obtained from the composite-plane acquisition using the reduced grid is contrasted with that obtained from the single-plane acquisition employing a full, regularly sampled grid. This result highlights the effectiveness of the proposed approach in maintaining spectral fidelity while significantly reducing the number of required sampling points.

However, it is interesting to perform a more detailed analysis for the technique, so the principal cuts for the PWS obtained from single-plane and composite-plane reduced acquisition are presented. In this case, the effect of introducing or not introducing the OTS coordinate correction will also be shown.

**Figure 14:**
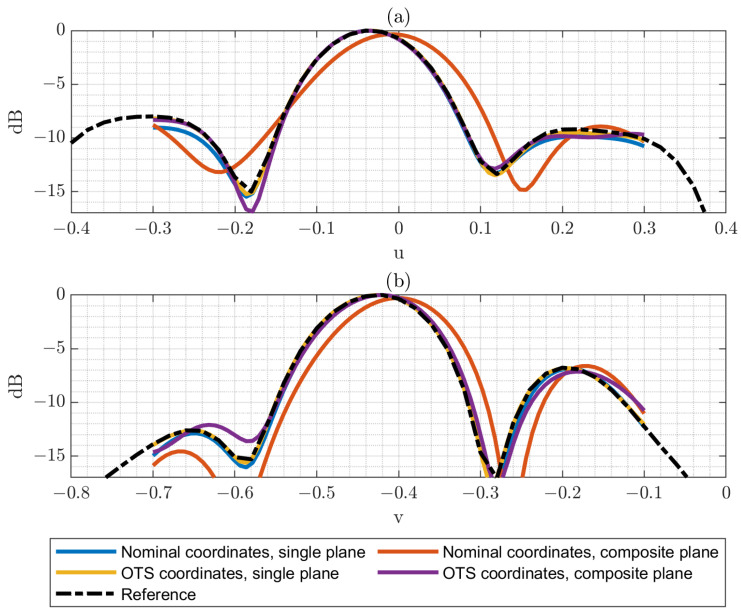
(**a**) Horizontal cut of the PWS (dB) obtained from both single-plane and composite-plane reduced acquisitions, with and without the application of optical correction. (**b**) Vertical cut of the PWS (dB) obtained from both single-plane and composite-plane reduced acquisitions, with and without the application of optical correction.

Figure 14 illustrates the results obtained with the optimized grid. From this figure, relevant conclusions can be drawn. First, in the single-plane case, the difference between applying or omitting the optical correction is relatively minor. This observation is consistent with the measured positioning errors, which remain below λ/10 across nearly the entire acquisition surface. Furthermore, the correspondence between the reduced grid and the full grid is notably good, confirming the robustness of the reduced sampling strategy in this configuration. Quantitative results can be seen in Table 4.

In contrast, for the composite-plane case, the impact of applying OTS correction is much more pronounced. The composition of multiple planes introduces a tilt in the reconstructed PWS, which originates from the positioning inaccuracies at the edges of the planar surfaces handled by the collaborative robot. These inaccuracies accumulate in the junction regions between adjacent planes, thereby affecting the overall reconstruction. Nevertheless, this distortion can be substantially mitigated by incorporating the OTS coordinate correction during post-processing, as clearly observed in the figure.

From these results, it can be concluded that the two techniques, the composite-plane approach and the reduced grid technique, can be effectively combined, even in scenarios where relatively large positioning errors have been intentionally introduced.

It is also important to assess the expected measurement time and computational cost of the proposed technique. Thus, the required times for the studied cases are summarized below.

The complete single-plane acquisition (measurement area of 700 mm × 900 mm regularly sampled, i.e., 71 × 91 points sampled each 10 mm at 10 GHz) requires 3.21 h, whereas the corresponding reduced grid (12 × 12 points) requires only 24 min (including both orthogonal polarizations in each case).

When the single plane is divided into individual planes for the composite-plane approach, the measurement time per individual plane scales proportionally. Therefore, each of the 700 mm × 700 mm individual planes employed in the 2 × 2 composite-plane, regularly sampled, requires 2.5 h (a total of 10 h). The corresponding 12 × 12 reduced grid is divided into four 6 × 6 individual planes, yielding an acquisition time of 3 min per plane (a total of 12 min).

However, additional times must be considered for robot repositioning and for the system calibration needed to correct human-induced repositioning errors. Repositioning the robot takes approximately 1–2 min plus an additional 1.5 min for the calibration procedure per individual plane. Total added time for a 2 × 2 composite plane, considering that the robot must be manually displaced three times, is therefore approximately 10 min.

Furthermore, the computational cost of the proposed method for computing the reduced grids must also be considered. The processing time for the computation of the studied reduced grids, using a laptop with an Intel^®^ Core™ Ultra 7 155H processor and 32 GB of RAM (Intel, Santa Clara, CA, USA), did not exceed 3–4 min for any of the cases.

Therefore, for the single plane example the acquisition time for the reduced grid is 24 min versus the 3.21 h required for the uniformly sampled grid, which corresponds to an approximate 88% reduction in time. For the 2 × 2 composite plane, the complete process for the reduced grid with a 2 × 2 composite plane takes approximately 38 min (i.e., 24 min + 10 min + 4 min) versus 10.23 h (10 h + 10 min + 4 min) for the uniformly sampled grid, corresponding to an approximate 94% reduction in time. These results represent a substantial improvement in both cases. However, it is worth remembering that the size of the 2 × 2 composite plane has been intentionally increased in order to analyze the effect of the positioning errors in the “worst-case” scenario, and it could be reduced to the size of the single-plane acquisition for a fairer comparison.”

## 5. Discussion

In this work, two validated techniques have been studied in combination in order to explore their mutual limitations and potential benefits. When used together, the composite-plane approach and the reduced-grid technique represent a powerful tool for in situ antenna measurements, as they enable the acquisition of a large effective area with portable equipment while significantly reducing the measurement time.

To provide context, the two methodologies were first summarized, highlighting their principles and how their performance can be jointly evaluated. Subsequently, general numerical examples were performed as a function of λ to avoid restricting the conclusions to a single operating frequency. Different reduced grids and acquisition errors were introduced, and their impact was systematically analyzed. By weighting the errors, both acquisition inaccuracy and imperfect plane composition were studied, the latter becoming more relevant as the error magnitude increased. The numerical results demonstrated that the two techniques can work together with satisfactory performance.

For the experimental validation, a 10GHz reflectarray antenna was employed as the AUT. A reduced grid was computed for this case, and a comparison between single-plane and composite-plane acquisitions was carried out. This evaluation included both complete and reduced grids, thereby allowing all possible combinations to be assessed.

Overall, the results presented in this paper are considered satisfactory. The observed errors are within acceptable limits, especially considering that the proposed measurement system is not intended for high-accuracy characterization, but rather for portable, in situ antenna measurements. Furthermore, all results were obtained in a non-anechoic environment, which introduces additional challenges but also demonstrates the practical applicability of the proposed methodology.

## Figures and Tables

**Table 1:** η (in %) from obtained PWS when introducing ϵ=λ/8 error distribution (with respect to the reference measurement).

	N = 6	N = 9	N = 12	N = 54
No OTS correction	2.448	2.433	3.698	2.196
OTS correction	0.499	0.094	0.003	0.001

**Table 2:** η (in %) from obtained PWS when introducing ϵ=λ/4 error distribution (with respect to the reference measurement).

	N = 6	N = 9	N = 12	N = 54
No OTS correction	9.363	9.608	15.903	9.388
OTS correction	0.548	0.094	0.004	0.002

**Table 3:** η (in %) from obtained PWS when introducing ϵ=λ/2 error distribution (with respect to the reference measurement).

	N = 6	N = 9	N = 12	N = 54
No OTS correction	41.61	40.60	72.37	43.13
OTS correction	0.648	0.095	0.006	0.002

**Table 4:** η (in %) from PWS rom the reduced grids (with respect to the reference measurement).

	N = 12
Nominal coord., single-plane	0.255
Nominal coord., composite-plane	12.90
OTS coord., single-plane	0.001
OTS coord., composite-plane	1.679

## Data Availability

The dataset accompanying this article includes the reduced-fields data from Section 4, the corresponding acquisition coordinates, and the reference PWS employed for comparison.

## Abbreviations

The following abbreviations are used in this manuscript:

UAV	Unmanned aerial vehicle
OTS	Optical tracking system
PNF	Planar near-field
SVD	Singular value decomposition
AUT	Antenna under test
IR	Infrared
OEWG	Open ended waveguide
PWS	Plane wave spectrum
